# Dispersion of pollutants in a porous medium with finite thickness and variable dispersion coefficients

**DOI:** 10.1016/j.heliyon.2022.e10083

**Published:** 2022-08-15

**Authors:** Calvia Yonti Madie, Fulbert Kamga Togue, Paul Woafo

**Affiliations:** a Laboratory for Environmental Modeling and Atmospheric Physics, University of Yaounde 1, P.O. Box 812 Yaounde, Cameroon; b Institute of Fisheries and Aquatic Sciences at Yabassi, University of Douala, Box 2701, Douala, Cameroon; c Laboratory on Modeling and Simulation in Engineering, Biomimetics and Prototypes, University of Yaounde 1, Box 812, Yaounde, Cameroon

**Keywords:** Groundwater, Pollutants, Boundary conditions, Contaminant transport, Dispersion coefficient

## Abstract

Groundwater is subject to the intrusion of pollutants of various types. These pollutants can have natural or anthropogenic sources. Their consumption can therefore affect human health, but also affects the development of vegetation. The objective of this article is to analyze the effect of the dispersion coefficient parameters on the spatio-temporal distribution of pollutants in a saturated porous medium with a finite thickness. This layer is subjected to two types of conditions at the outlet of the aquifer where the dispersion coefficient is a function of depth. The partial differential equations were solved using an implicit finite difference technique. The results of the analysis suggest that the behavior of the concentration profile was influenced by the different output boundary conditions. On the other hand, the parameter, b of the dispersion coefficient depending on the distance has a significant impact on the movement of the solute in a saturated porous medium compared to the parameter, a. In other words, it has more effect on the dispersion of pollutants in aquifers. This study highlights the need to bring insight on the transports parameters while modeling the transport of solute in a porous media.

## 1 Introduction

The problem of the dispersion of solutes during the movement of fluids was at the center of interest at the beginning of this century, but it was not until 1905 that the general theme of hydrodynamic dispersion or miscible displacement became one of Ravi's most systematic studies (2014); Patel et al. (2014). The mathematical statement of the solute transport equation, also called the advection-dispersion equation, has been accepted as a model to describe the migration and position of solutes in groundwater Patel et al. (2014); Xie et al. (2019). For a particular case, the equation includes evolutionary and dispersive transport processes. Advection-dispersion equation models are used to describe the movement of solutes in porous media and also allow for the rapid location of water potability sites in these media. Analytical and numerical solutions of these, generated by initial boundary conditions, remain an essential tool for environmentalists, hydrologists, civil engineers, and mathematical modelers to examine the process of remediation and management of a contaminant in a hydro-environment and to validate their experimental work (Pickens and Grisak, 1981).

The use of the coefficient of dispersion of pollutants in groundwater has been discussed by many researchers from different angles. Here are some examples. Depth-dependent dispersion was experimentally justified by Pickens and Grisak, 1981, as he reorganized several transport assays from fields and laboratories to predict the transport of contaminants in heterogeneous porous media. The accuracy and capability of the scale-dependent dispersion model in a heterogeneous medium was simulated by Pickens and Grisak, 1981 across heterogeneous soil using the values of the asymptotic dispersivity, a = 20 m and the characteristic half of its length, b = 50 m. Hamza (2003) relies on a particular solution derived from various diffusion functions and mass injection scenarios which includes the linear, asymptotic and exponential variation of the diffusion functions to evaluate the migration of the solute with a time-dispersion dependent coefficient with the three scenarios.

Djordjevich and Savovic´ (2013) used the implicit finite difference method to solve the advection-dispersion equation in their work. This method is unconditionally stable and yet it does not result in higher computational efficiency because extremely large matrices have to be manipulated at each computational step. The finite difference method is also easier to program in addition to being more computationally efficient Savovic´ and Djordjevich (2013).

Other researchers have used the term speed-dependent dispersion to assess numerically using a specific technique based on an adaptive scheme. They also showed that the numerical integration and the nonlinear dependence of the dispersion with respect to velocity make the semi-analytical solution impractical Fahs et al. (2016). Abgaze and Sharmar (2015) present in the first part of their study, constant, linear and asymptotic dispersion coefficients as a function of distance to describe the scaling effect and to simulate the experimental fracture curves observed in a column and simulate experiments with long, thin soils. A comparative study was carried out between distance-dependent dispersion and constant dispersion, simulating experimental data on the transport of solutes in the soil's column with a constant mass transfer coefficient. Yan et al. (2015) proposed an empirical correlation describing the interaction of asymptotic behaviors of longitudinal and transverse dispersion of the solute for flow through packets of random particles. Dagan (1984) used a similar approximation by satisfying a diffusion equation with time-dependent apparent dispersion coefficients to determine the expected value of the concentration. Lee et al. (2018) used the meaning of local equilibrium when examining with local equilibrium to model longitudinal dispersion in groundwater. Silliman and Simpson (1987) performed laboratory work using various tests to assess changes in dispersivity caused by the presence of heterogeneities. Molz et al. (1983) relied on the definition of a scale-dependent macro dispersion coefficient to study a unidirectional flow in a stratified aquifer, then obtained for the macro-dispersivity the different components within a limited time.

A semi-analytical solution of the transport model with an asymptotic dispersion depending on the distance and on the asymptotic dispersivity parameters (a), and on the half-length characterizing the mean stroke (b), was developed by Sharma and Abgaze (2015) taking into account a condition of zero pollutant concentration at the outlet of the domain and parameters of constant asymptotic dispersivity in a homogeneous porous medium. This author neglected certain boundary conditions that may occur at the aquifer boundary due to natural phenomena, as mentioned in the works of Al-Niami and Rushton, (1976); Yadav et al. (2012) and the variability of the asymptotic dispersivity parameters in a heterogeneous porous medium.

In our recent study Madie et al. (2022a), the advection dispersion equation was solved numerically to evaluate the concentration profile of salinity in aquifers for a distance-dependent dispersion coefficient problem. A distance-dependent adsorption coefficient and a constant asymptotic dispersion parameters *a* and *b* were also considered. Three initials concentration injection condition were introduced at the inlet of the aquifer: (a) constants, (b) variables exponentially with time and (c) sinusoidally with time.

This article proposes a numerical solution of the transport model with an asymptotic dispersion dependent on the distance with the parameters of asymptotic dispersivity (a), and the half-length characterizing the mean stroke (b) developed by Sharma and Abgaze (2015), by taking two conditions at the outlet of the domain and a variable asymptotic dispersion parameters *a* and *b* in a heterogeneous porous medium.

Our objective is to examine the impact of the asymptotic dispersion parameters on the spatio-temporal variation of pollutant concentration in the underground environment.

## 2 Materials and methods

### 2.1 Description of the physical model

**Figure 1:**
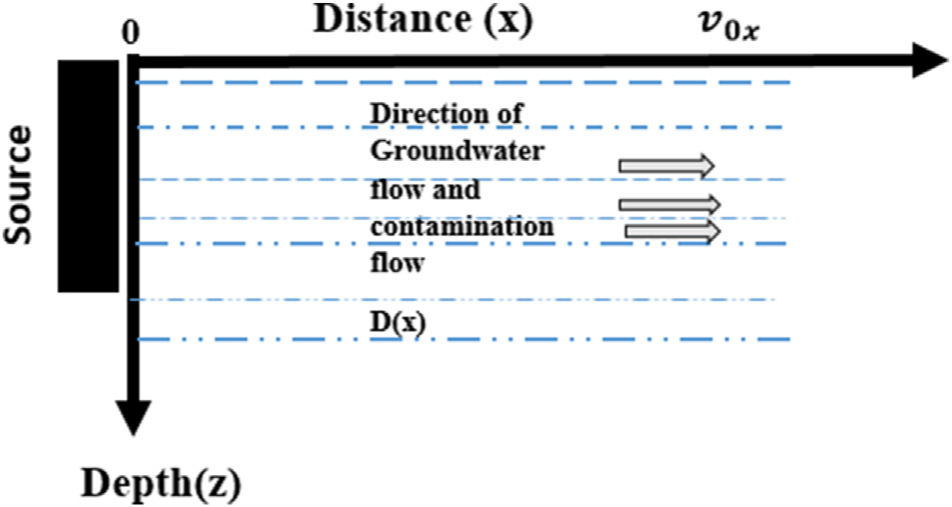
Physical model of the system.

Figure 1 illustrates the physical model of the problem. In this model, dispersion coefficient varies with respect to the depth, x, one-dimensional and the Darcy velocity is constant. The boundary conditions are defined at the boundaries of the matrix.

### 2.2 Mathematical description

The heterogeneity of most geological materials is caused by certain natural deformations, such as fractures, and lenses of high or low permeability which are at the origin of the variation of the hydraulic properties and the spatial or temporal increase of the dispersivity in the porous medium (Kangle et al., 1996; Madie et al., 2022b). Most geological materials are extremely inhomogeneous due to the presence of irregular stratifications, cracks and fractures and lenses of high or low permeability. These non-homogeneities cause the hydraulic properties to vary in space, leading to fluctuations in the speed of the fluid, and eventually give rise to a dispersivity which increases with distance or time. Field and experimental evidence from studies has been suggested by Pickens and Grisak, 1981 that the dispersion coefficient is not constant but apparently increasing with time displacement and distant traveled by the solute. The phenomenon of dispersion is a phenomenon which moves away from the place of injection, the mass of solute is diluted during its displacement along the direction of flow x to occupy an increasing volume with a relatively decreasing concentration. The latter suggests that in a heterogeneous medium the pollutants transport equation taking into account a constant dispersion parameter become unsuitable, for this reason the distance-dependent dispersion coefficient is applied in the advection-dispersion equation below Madie et al. (2022a).

The transport equation, defining the variation in concentration as a function of time and space of a dissolved substance transferred into a heterogeneous saturated porous medium, is written as the linear Eq. (1) form Sharma (2015); Yadav (2018):

(1) $$R\frac{\partial c}{\partial t}=D\left(x\right)\frac{\partial^{2}c}{\partial x^{2}}-v_{0x}\frac{\partial c}{\partial x}$$

This equation involves two major terms influencing the transfer potential of the solute through a saturated porous medium. *c* is the concentration of the pollutants, R a delay factor generated by the absorption of the pollutant, t time and x distance traveled by the fluid in the porous medium. Convection or fluid flow velocity ($v_{0x}$) describes the transport of a solute by the movement of a fluid moving through a porous medium Gao et al. (2010). This water flow in a saturated porous medium (absence of gas in the pores) is defined by the following relation: $v_{0x}$ = q/θ or q = ki (Darcy flow) and θ: the effective porosity of the medium; k: the permeability of the aquifer, i: the hydraulic gradient of the aquifer (Figure 1).

### 2.3 Physical description of the distance-dependent asymptotic dispersion coefficient parameter

The variation of many subsurface parameters caused by natural disaster makes it difficult to use the dispersion-advection equation with a single parameter to predict the concentration of contaminants at different distances in a porous medium. The asymptotic dispersivity is a physical quantity considered to account for the heterogeneity of porous media. Asymptotic dispersivity, which depends on the distance travelled by the fluids, is used to define the notion of a distance-dependent dispersion model for solute transport in heterogeneous porous media (Kangle et al., 1996; Madie et al., 2022a). The hydrodynamic dispersion coefficient D (x) which is a function of the distance in the homogeneous porous medium in the absence of any molecular diffusion due to very fine tortuosity is written as Eq. (2) below:

(2) $$D\left(x\right)=D_{0}+\alpha \left(x\right)v_{0x}$$

where α (x) is the dispersivity and $D_{0}=\left[D_{e}\tau \right]$ is the molecular diffusion caused by molecular motion and particle collisions; with *τ* ≤ 1: tortuosity of the porous medium, $D_{e}$ is the effective dispersion. Asymptotic dispersivity is responsible for the heterogeneity of porous media and it initially increases with the distance traveled and eventually approaches an asymptotic value, Yan et al. (2015). Since the asymptotic dispersion coefficient depends on the distance, such a relation can be expressed Eq. (3) below:

(3) $$D\left(x\right)=D_{0}+a\left(1-\frac{b}{x+b}\right)v_{0x}$$

where a is the value of the asymptotic dispersivity and b is a characteristic distance which determines the distance of displacement for the dispersivity to reach half of the asymptotic value. Dispersivity is the ratio of the asymptotic dispersion coefficient to the pore velocity of water. The values of b depend on the extent of the pre-asymptotic zone. For a smaller value of b, the dispersivity will approach the asymptotic value more quickly. The accuracy and capability of the scale-dependent dispersion model in a heterogeneous medium was simulated by Pickens and Grisak (1981) across heterogeneous soil using the experimental values of the asymptotic dispersivity, a = 20 m and the characteristic half of its length, b = 50 m and no molecular diffusion.

### 2.4 Initial and boundary conditions

We consider a horizontal unidirectional flow of water through an aquifer of finite length L, with an initial concentration $c_{0}$ at the inlet of the aquifer, the movement is rather in the direction of the flow x. The concentration water infiltrates into the soil matrix, then, influenced by the parameters of the soil, and becomes a source of drinking water at zero concentration at the arrival of the boundary for L infinite Madie et al. (2022a). The problem can be defined mathematically by Eq. (1) with the following initial and boundary conditions given by Eq. (4):

(4) $$\left\{ \begin{array}{c} c\left(x,0\right)=0;for\;0<x<L\\ c\left(0,\;t\right)=c_{0}\;for\;all\;t,\\ c\left(L,t\right)=0\;for\;t>0 \end{array}\right.$$

The boundary condition of Eq. (4) assumes that the concentration at (x = L) is held to zero at all times as shown by the work of (Sauty (1980); J. W. Biggar and Nielsen (1971), this may not always happen in practice. An alternative condition which allows a certain dispersion to occur at the boundary is given by Eq. (5):

(5) $$\left\{ \begin{array}{c} c\left(x,0\right)=0;for\;0<x<L\\ c\left(0,\;t\right)=c_{0}\;for\;all\;t,\\ \frac{\partial c}{\partial x}\left(L,t\right)=\frac{v_{0x}}{2D\left(x\right)}c\left(L,t\right)for\;t\geq 0 \end{array}\right.$$

The boundary condition of Eq. (5) assumes that the amount of pollutants crossing the boundary (x = L) is proportional to the flow producing at the boundary because the accumulation time has not yet arrived, hence the accumulation term of Eq. (1) canceled out ($\frac{\partial c}{\partial t}=0$) to give rise to this flow. This phenomenon of proportionality at the boundary occurs in practice when poor quality water is prevented from spreading by a flow of fresh water. In practice, the flow is reduced due to natural causes or due to increased exploitation of good quality water and the risk of poor quality water spreading is very high Al-Niami and Rushton, (1976), Yadav et al. (2012).

### 2.5 Numerical solution of the mathematical model

Finite difference methods are generally used to solve fluid flow equations in porous media. The difference methods allow the calculation of the average load per mesh and provide the exact mass balance at each element. The discretization of the fluid flow equation by finite differences is very obvious and simple to implement, but applicable on rectangular or cubic meshes Wang and Anderson (1982). The derivatives of the fluid flow Eq. (1) are approximated by the finite difference numerical scheme to determine the first and second order spatial derivatives, which are defined from Eqs. (6) and (7) (Natarajan et al., 2020; Madie et al., 2022b):

(6) $$\frac{\partial c}{\partial x}=\frac{c_{i}^{j}-c_{i-1}^{j}}{\text{Δ}x}$$

(7) $$\frac{\partial^{2}c}{\partial x^{2}}=\frac{c_{i+1}^{j}-2c_{i}^{j}+c_{i-1}^{j}}{\text{Δ}x^{2}}$$

The first order temporal discretization is given by Eq. (8) written as:

(8) $$\frac{\partial c}{\partial t}=\frac{c_{i}^{j+1}-c_{i}^{j}}{\text{Δ}t}$$

The indices (i) and (j) indicate the nodes of discretization along (x) and (t) respectively. Δx and Δt are respectively the spatial and temporal steps. Thus, Eq. (1) can be written in the discrete form such as Eqs. (9) and (10):

(9) $$c_{i}^{j+1}-c_{i}^{j}=\frac{D_{i}\text{Δ}t}{R\text{Δ}x^{2}}\left(c_{i+1}^{j}-2c_{i}^{j}+c_{i-1}^{j}\right)-\frac{v_{0x\Delta t}}{R\text{Δ}x}\left(c_{i}^{j}-c_{i-1}^{j}\right),$$

(10) $$c_{i}^{j+1}=\left(1-2\propto -\beta \right)c_{i}^{j}+\propto c_{i+1}^{j}+\left(\propto +\beta \right)c_{i-1}^{j},$$

where $\propto =\frac{D_{i}\Delta t}{R\Delta x^{2}}$ and $\beta =\frac{v_{0x\Delta t}}{R\Delta x}$

The discretization of boundary and initial conditions is necessary to apply this method. The discrete version of the initial condition and boundary conditions given successively by Eqs. (4) and (5) is expressed as Eqs. (11) and (12):

(11) $$\left\{ \begin{array}{c} c_{i}^{0}=0,\;if\;0\leq i\leq Nx\\ c_{0}^{j}=c_{0},\;if\;j\geq 0,\\ c_{Nx}^{j}=0,\;if\;j>0 \end{array}\right.$$

(12) $$\left\{ \begin{array}{c} c_{i}^{0}=0,\;if\;0<i<Nx\\ c_{0}^{j}=c_{0},\;if\;j\geq 0,\\ \frac{\partial c_{Nx}^{j}}{\partial x}=\frac{v_{0x}}{\;2D_{i}}c_{Nx}^{j}\;,if\;j\geq 0 \end{array}\right.$$

These two relationships express a column without contamination at the initial moment where c_o_ is the concentration of pollutants at the entrance to the aquifer.

### 2.6 Stability conditions

An approach similar to that used by Natarajan et al., 2020 where the spatial and temporal steps tend to zero, which also leads the error to tend to zero is used to obtained stability conditions. This leads to imposing certain conditions on the finite difference scheme of the discrete Eq. (10) which are given by the following Eq. (13):

(13) $$\left\{ \begin{array}{c} 0\leq 1-2\propto -\beta \leq 1\\ 0\leq \propto \leq 1,\\ 0\leq \propto +\beta \leq 1 \end{array}\right.$$

The new solution is a convex combination of the solution at new time step (j + 1) at a spatial node i is an average of the solutions at the previous time step at the nodes i − 1, i and i + 1. The new solution linked continuously to the initial value of $c_{i}^{0}$. This implies

(14) $$\left\{ \begin{array}{c} \beta \leq 1-2\propto \leq 1+\beta \\ 0\leq \propto \leq 1,\\ -\propto \leq \beta \leq 1-\propto \end{array}\right.$$

By simplifying Eq. 14a and 14c, we have −∝≤ β ≤ 1 − 2∝

Thus, the stability conditions are 0≤ ∝ ≤1 and −∝ ≤ β ≤ 1 − 2∝.

In this work $\;0\leq \frac{D_{i}\Delta t}{R\Delta x^{2}}\leq 1\;$ and $-\frac{D_{i}\Delta t}{R\Delta x^{2}}\leq \frac{v_{0x\Delta t}}{R\Delta x}\leq 1-2\frac{D_{i}\Delta t}{R\Delta x^{2}}$. The data in Table 1 were taken from the work of Al-Niami and Rushton (1977); Pickens and Grisak (1981).

**Table 1:** Parameter values used to simulate concentration profile.

Parameters	Symbols	Values
Asymptotic dispersivity value	a	20 m
Characteristic distance	b	50 m
Flow velocity	v	0,8 m/d
Retardation factor	R	2.5

## 3 Results and discussion

### 3.1 Validation of the model

**Figure 2:**
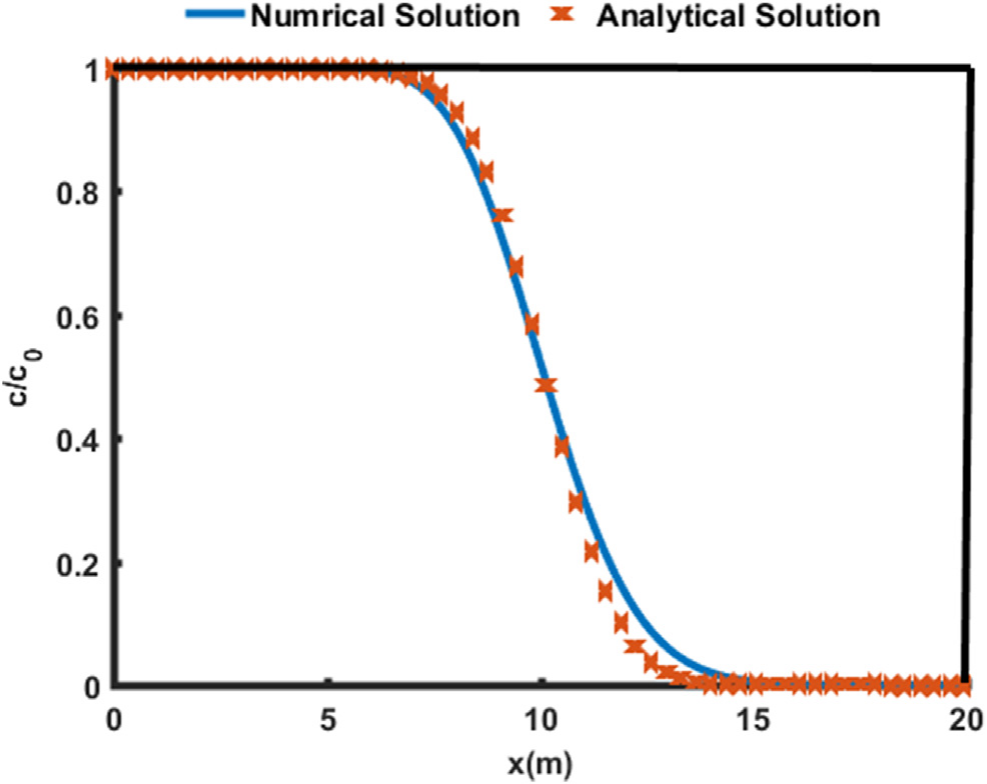
Validation of the numerical solution with analytical solution of the one-dimensional advection-dispersion equation in a homogeneous medium provided by Ogata and Banks (1948). Parameters used: speed = 1 m/d; dispersion coefficient = 0.1 m^2^/d; delay factor = 1; total duration of the simulation = 10 days; and domain length = 100 m.

An analytical solution provided which constant coefficient by Ogata and Banks (1948) is used as shown in Figure 2 to justify the correct implementation of the model. It is observed from Figure 2 that there exists a close agreement between the numerical solution and the analytical solution. Therefore, our model can be used to predict the concentration distribution curves as a function of the thickness of the aquifer with different entry and exit boundary conditions.

### 3.2 Variation of pollutants concentrations in the system

In this section the discrete Eq. (10) obtained by the finite difference method associated with the initial and boundary conditions of the discrete Eqs. (11) and (12) is solve numerically to obtain the results below.

The numerical solutions of the advection dispersion Eq. (1) is evaluated as a function of aquifer length, time, dispersion coefficient varying as a function of distance traveled and the rate of flow of fluids in the aquifer. For this purpose, the model parameters used are presented in Table 1, were selected in previous studies Guangyao Gao., (2010); Kangle et al. (1996); Wang et al. (2020); Natarajan et al., 2020; N. Natarajan (2015); Al-Niami and Rushton (1976).

The first condition represents the boundary condition presented in Eq. (4) and the second condition is the boundary condition presented in Eq. (5).

**Figure 3:**
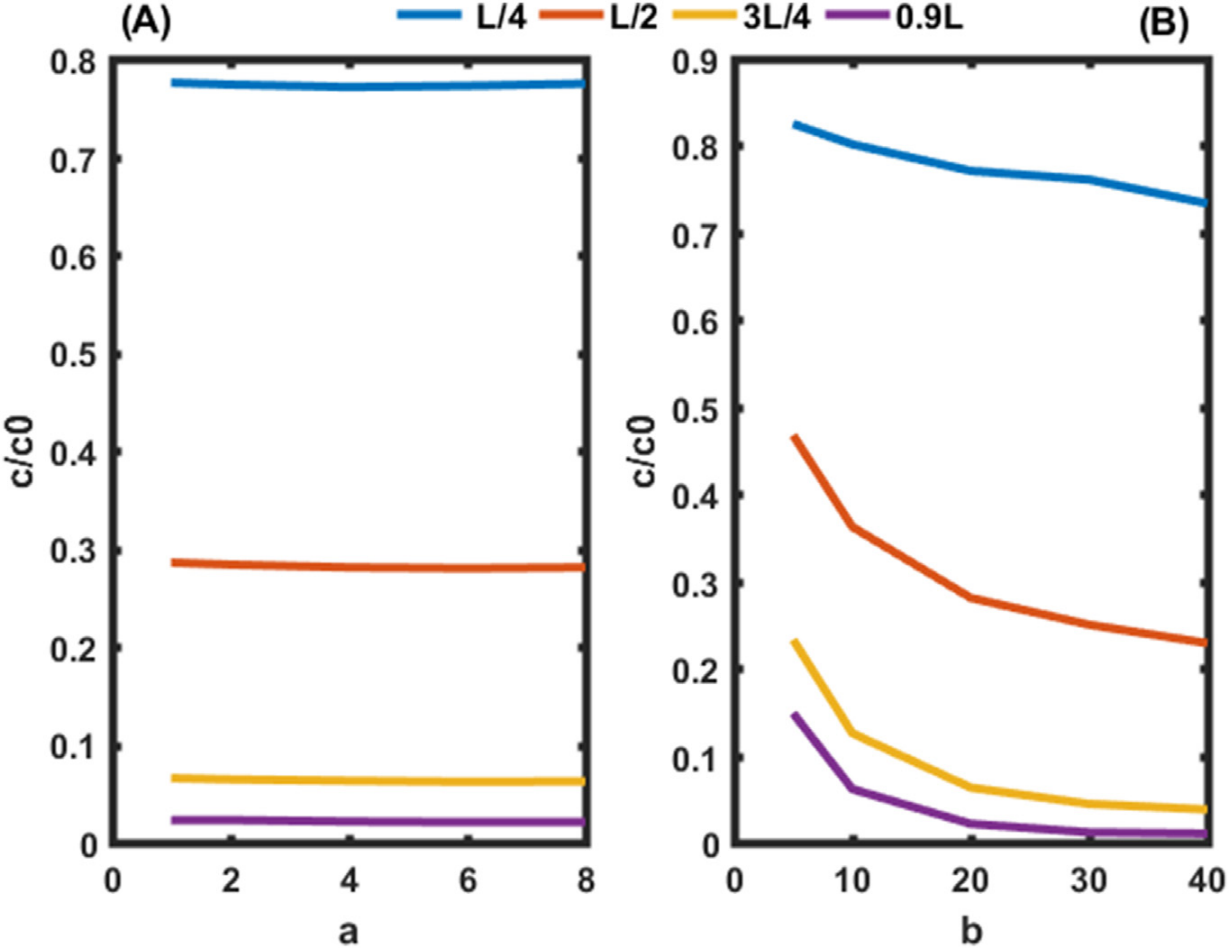
Variation of pollutants concentrations in the system as function of *a* and *b.* In (A) initial and boundary conditions given by the first condition (t = 50 day, R = 2.5, v = 0.8 m/d,a = 8 m). In (B) initial and boundary conditions given by Eq. (7) (t = 50 day, R = 2.5, v = 0.8 m/d, b = 40 m^−1^).

**Figure 4:**
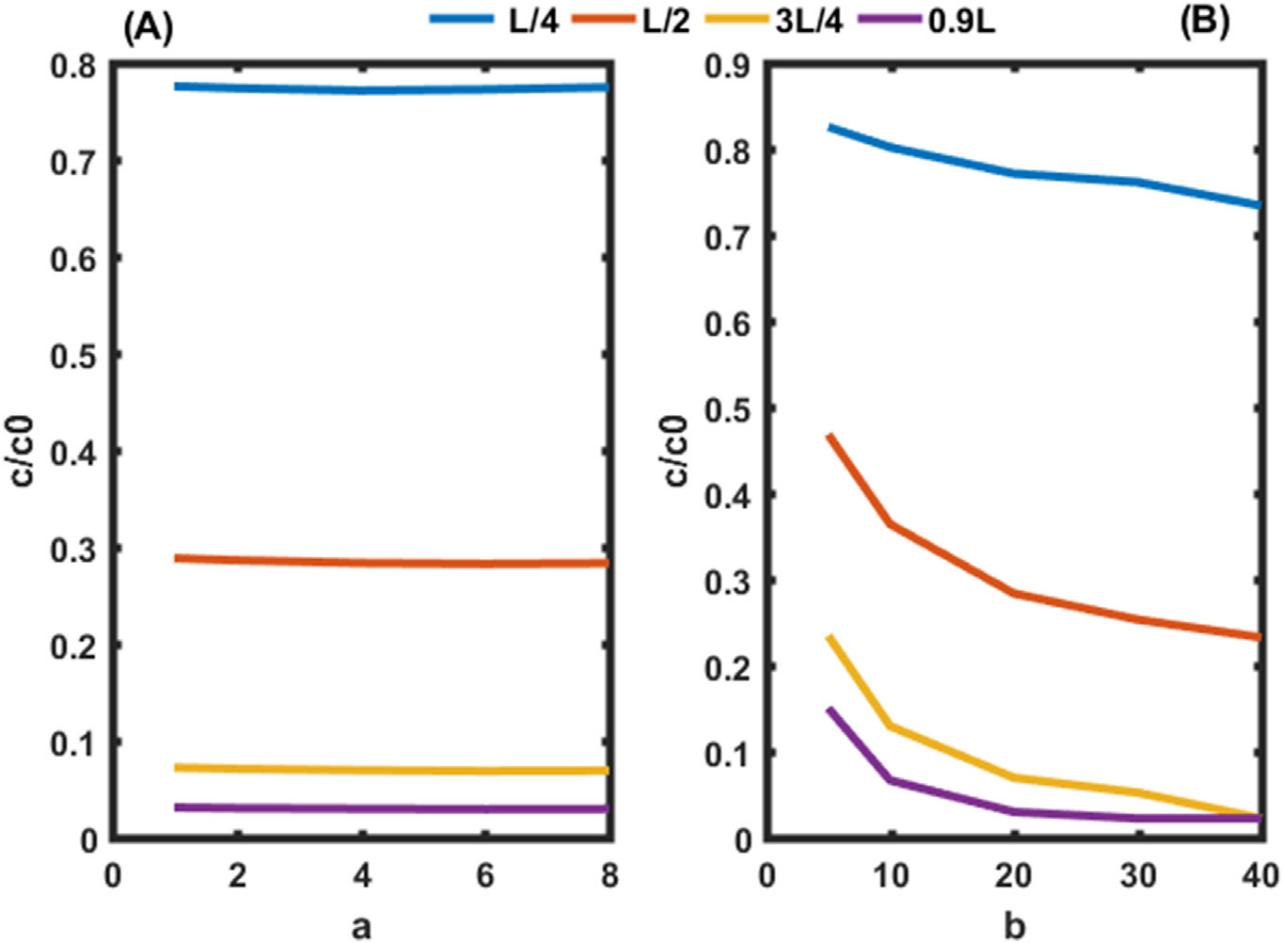
Variation of pollutant concentrations in the system as a function of a and b. In (A) initial and boundary conditions given by the second condition (t = 50 day, R = 2.5, v = 0.8 m/d,a = 8 m). In (B) initial and boundary conditions given by Eq. (8) (t = 50 day, R = 2.5, v = 0.8 m/d, b = 40 m).

Four points were selected in the aquifer (L/4, L/2, 3L/4, 0.9L) to assess the behavior of the dispersion of pollutants in the system. Figures 3 and 4 show the relationship between the evolution of the concentration of pollutants at different points and the dispersion coefficients parameters a and b using the initial and boundary conditions given respectively by Eqs. 4 and 5. Higher values of pollutant concentrations are obtained near the source (x = L/4) while low values were found near the outlet of the system (x = 0.9 L). These results show that the dispersion of pollutants increases with the distance of transport. Figures 3 and 4 inform us about the points that were exploited by Sharma and Abgaze. (2015) from the entrance to the exit of the aquifer, because in their work they considered the value a = 0.8273 m and b = 1.4821 m. Chen et al. (2008) as from them, they rather fix the value a = 0.284 m and b = 10 m. In the light of Figures 3 and 4, it appears that the data for the values of a and b used in these cited work are well located in a specific zone of the aquifer according to the selected points. The results also suggest that the parameter b has an important effect on the dispersion of pollutants (Figure 3B) rather than on the asymptotic dispersivity a (Figure 3A).

**Figure 5:**
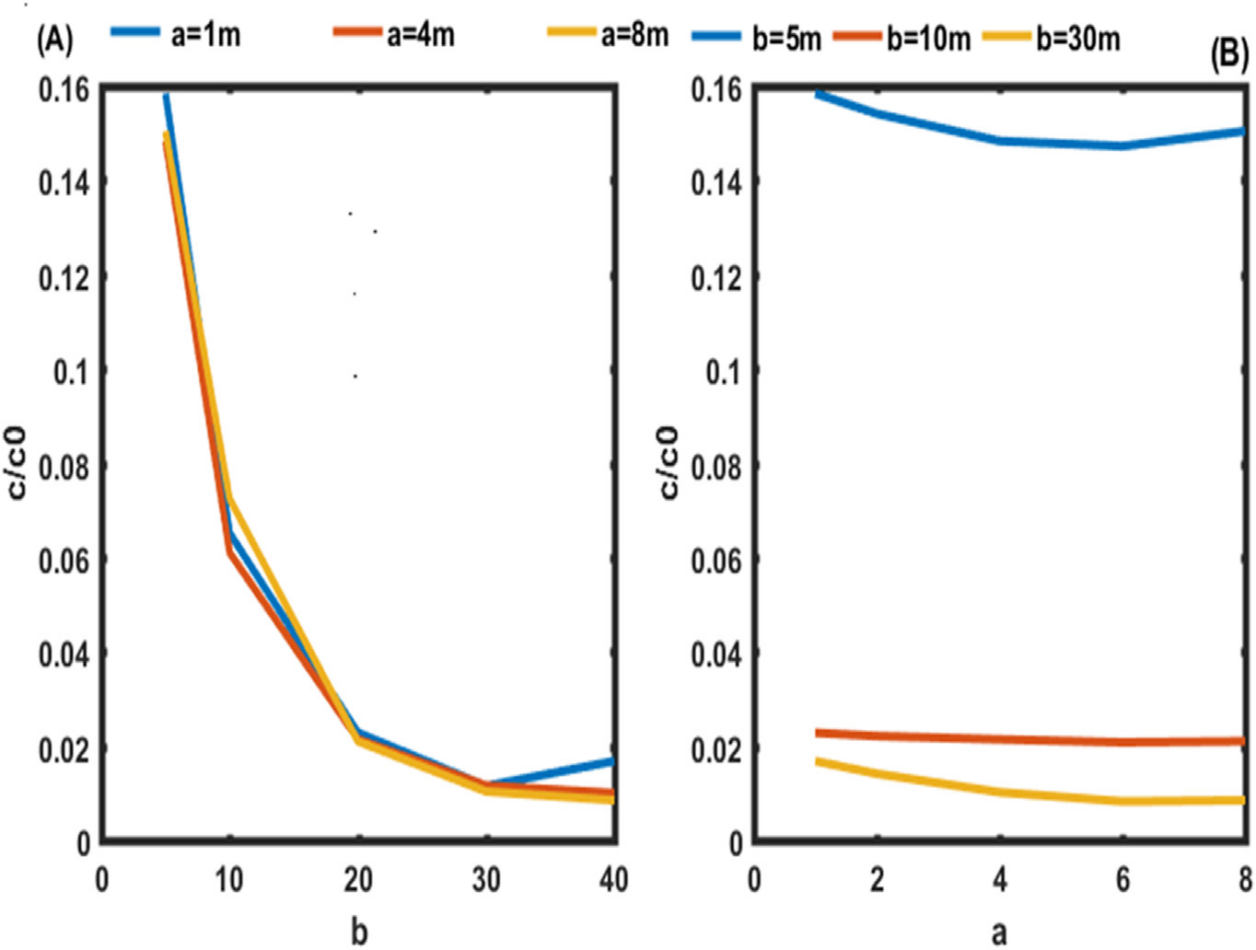
Behavior of pollutants on leaving the aquifer (0.9L) for the first condition. In (A) the behavior of pollutants is given in function of b. In (B) the behavior of pollutants is given in function of a.

The concentrations are higher in Figure 4 due to the initial and boundary conditions (Eq. 8). For proper environmental modeling of solute transport in porous media, experimental studies should be performed to find the values of parameters a and b rather than fixing them as is done in the literature Guangyao Gao., (2010); Kangle et al. (1996). It is also possible to find the range of these parameters which could improve the performance of the system (Figure 5).

**Figure 6:**
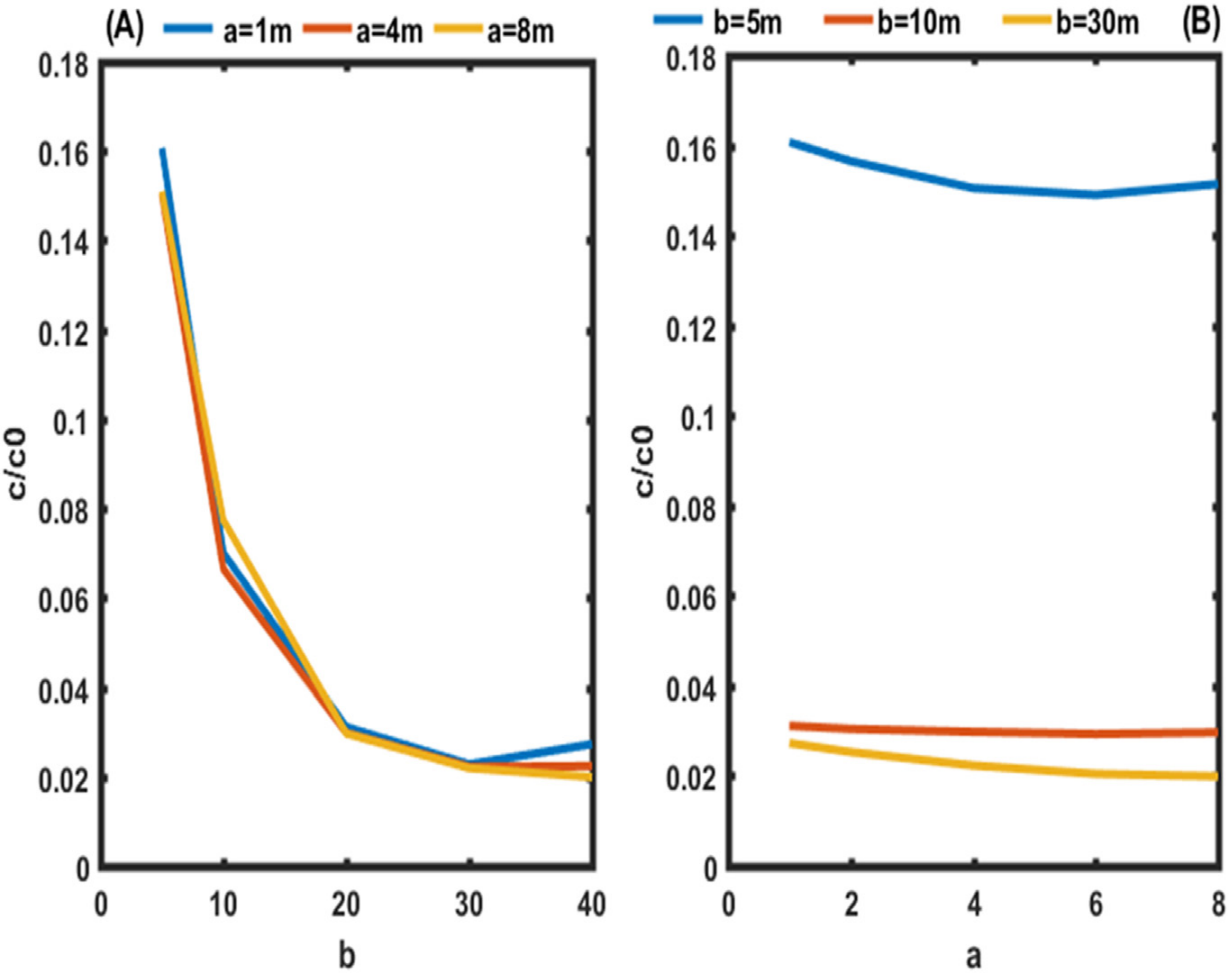
Behavior of pollutants at the outlet of the aquifer (0.9L) for the second condition. In In (A) the behavior of pollutants is given in function of b. (B) the behavior of pollutants is given in function of a.

Figures 5 and 6 show the evolution of pollutant concentrations as a function of the various constants of dispersion coefficients a and b near the outlet of the aquifer (0.9 L) for the two cases, where the constraints of the pollutants are zero (Figure 5) and not zero (Figure 6). These two Figures should allow us to select the exact values of a and b.

Figures 5(A) and 6(A) shows the spatial variation of the concentration of pollutants in the porous medium for different values of the dispersivity a (a = 4 m, 8 m, 12 m, 16 m, 20 m) of the dispersion function as a function of depth. Figures 5(A) and 6(A) shows that the mass of solute retained in the medium increases with the value of the dispersivity. The concentration profiles follow an exponential decrease in the aquifer independently of the value of the dispersivity a. This result is similar to those of Kangle et al. (1996). These authors analytically solved the one-dimensional dispersion advection equation to predict solute transport in heterogeneous porous media with scale-dependent dispersion. It appears that the mass of solute retained in the porous medium increases with the value of dispersivity a. It is also observed that the slope of the concentration profile becomes closer and closer with an increase in a. This is due to the asymptotic variation of the dispersion coefficient which approaches more and more its asymptotic value in the porous medium.

It was noted in Figure 5(B) that, for b equal to 5 m and for all the values of for a ∈R + in the aquifer, we observe high values of concentrations with a range between 14 and 15% of the initial concentration. It was also noted in Figure 5(B) that, for b equal to 40 m and for all the values of a ∈R + we observe the lowest values of concentrations with a range between 2 and 3% of the initial concentration. From the analysis of the dispersion constants a and b for the stresses of zero concentrations at the outlet of the aquifer, it emerges that the appropriate values of b are equal to or greater than 40 m for all values of for a ∈R +. High concentration values are obtained for b less than 5 m.

**Figure 7:**
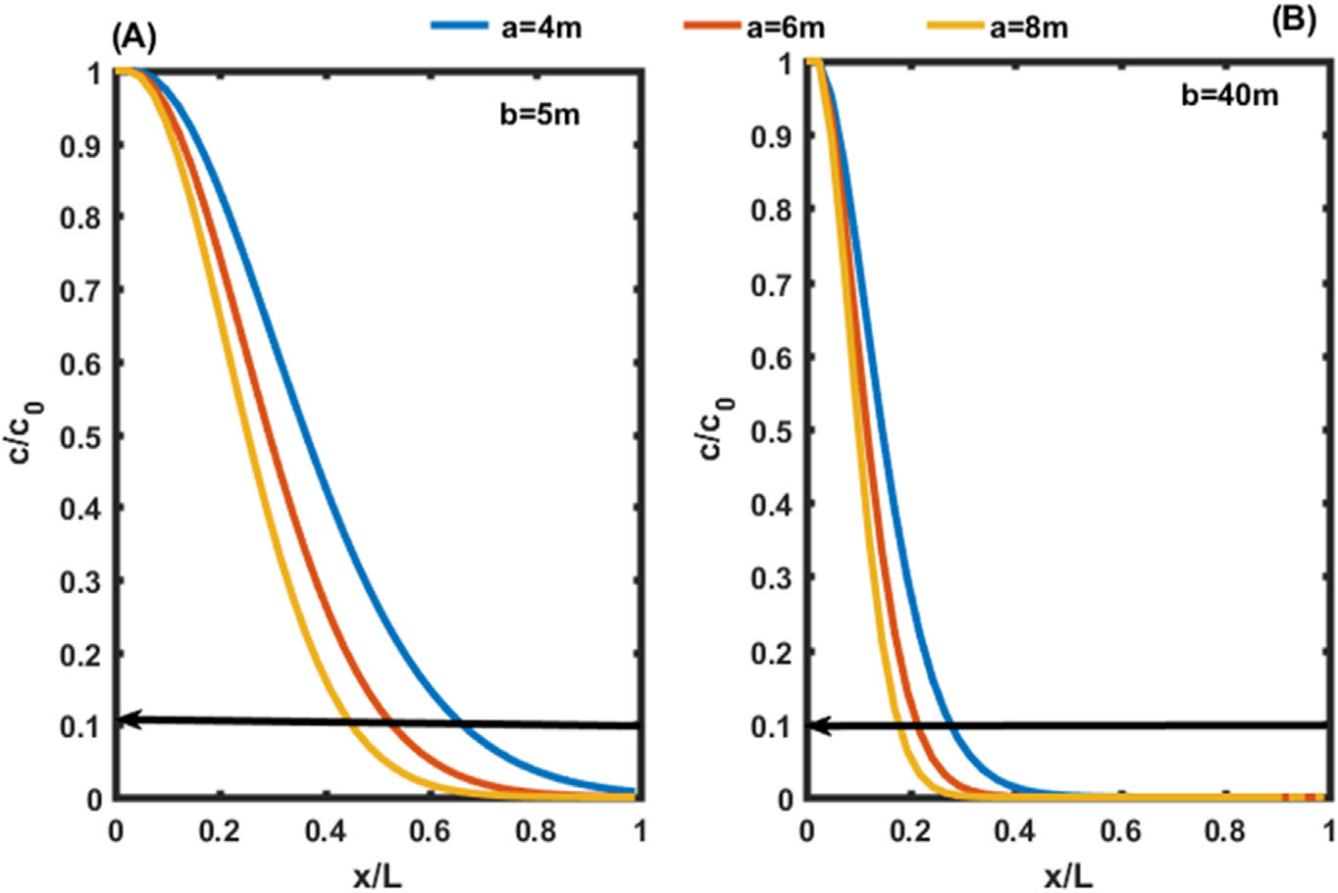
Concentration distributions as a function of distance at t = 50 days and v = 0.8m/day, for the first condition. In (A) the Concentration distributions is given for b = 5m. In (B) the Concentration distributions is given for b = 40 m.

Figure 6(B) shows that, for b equal to 5 m, the same trend is observed as in Figure 5(B) for non-zero concentrations at the outlet of the aquifer. However, for b equal to 40 m and for all the values of a ∈R + we observe the lowest values of concentrations with a range between 1 and 2% of the initial concentration rather than 2 and 3% as observed on Figure 5(B). These values of b found are used at different points of the aquifer in this work to analyze the effect of the dispersion coefficient as a function of distance and time on the transport of contaminants in porous media (Figure 7).

Figures 7(A) and 7(B) successively illustrate the spatial distribution of the concentration of pollutants with chosen constants dispersion coefficients b = 5 m and b = 40 m. An interesting observation from the graphs above is that the concentration profiles for the two values of b above follow an exponential decay pattern in the aquifer for any value of a (a = 4 m, 6 m, 8 m). These results are similar to those obtained by Kangle et al. (1996). These authors have developed a general analytical solution for one-dimensional solute transport in heterogeneous porous media with scale-dependent dispersion. The results obtained in Figures 7(A) and 7(B) show that by allowing 10% initial concentration (10% of C_0_) as a guide value in a porous medium, the suitable points of installation of drinking water points are in the order of x/L = 0.65; 0.53; 0.45 respectively for a = 4 m, 6 m, 8 m and b = 5 m. On the other hand, for b = 40 m, the order of these positions becomes x/L = 0.28; 0.21; 0, 17 respectively for a = 4 m, 6 m, 8 m. These positions decrease with *a* an increase in the value of *b*.

**Figure 8:**
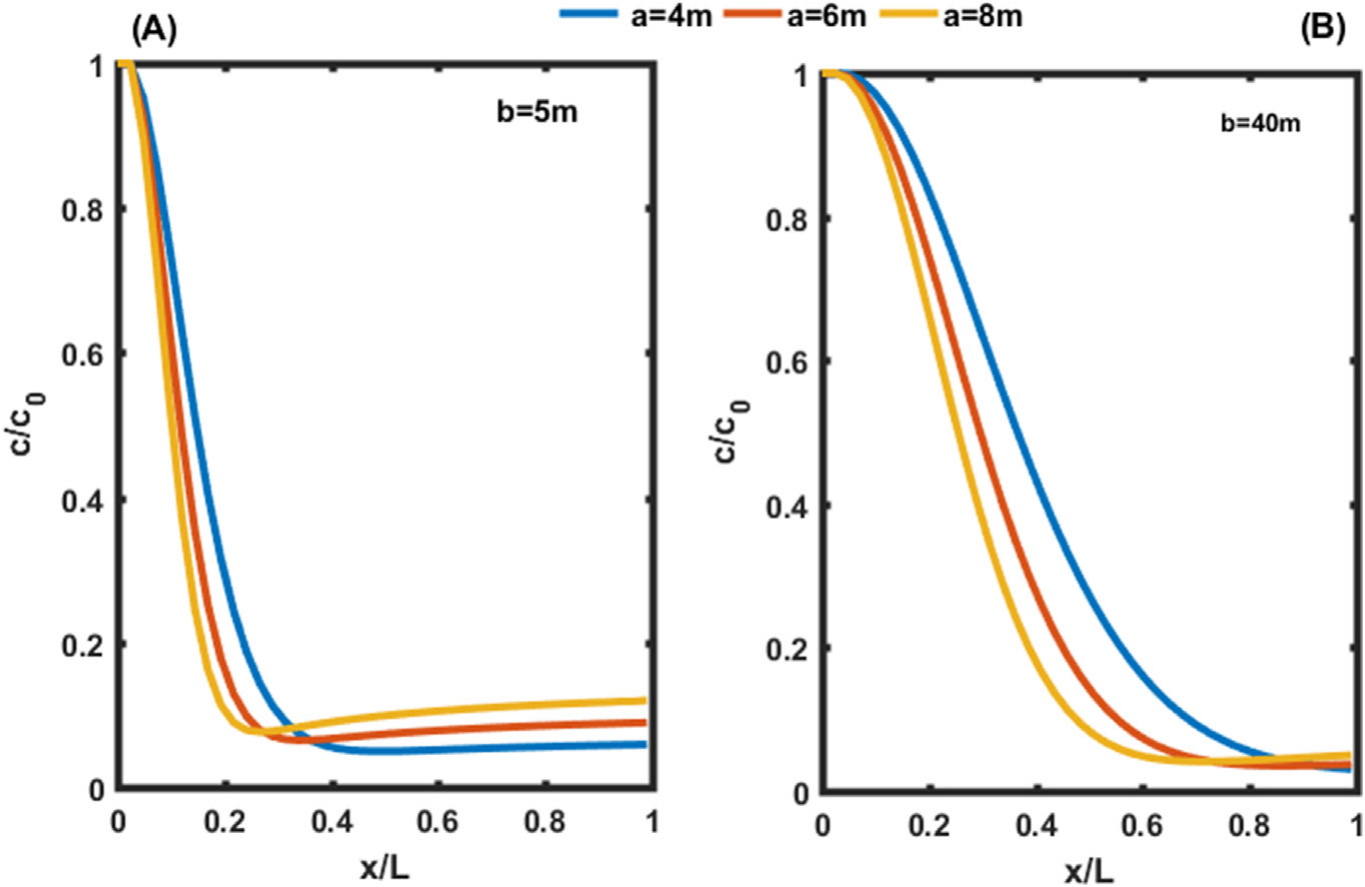
Concentration distributions as a function of distance at t = 50 days and v = 0.8m/day for the second condition. In (A) the Concentration distributions is given for b = 5m. In (B) the Concentration distributions is given for b = 40 m.

Figure 8 keeps the same behaviour as Figure 7 with the only difference that the results are different at the outlet of the aquifer due to the boundary condition imposed at the other end of the aquifer. This work revealed that the adsorption and transport of pollutants in heterogeneous underground environments are improved when the value of the parameter b increases. Thus, b has a strong effect on the transport of solutes in the aquifer. However, Williams et al. (2003) have demonstrated the significant effects of pH and temperature on transport through porous columns. Natural phenomena (precipitation and climate change) also influence the parameters of the subsoil (Vengadesan and Lakshmanan, 2019).

## 4 Conclusion

Groundwater is recognized as an economic resource and an ecological heritage that is important to manage and preserve. This is greatly important for a country located in coastal areas where the water requirements (irrigation and industry) are mainly groundwater. This article proposes the numerical resolution of the unidimensional advection-dispersion by the implicit finite difference technique method. The effect of dispersion, function of the distance of the transport of solutes in a porous medium has been analyzed for different boundary conditions at the outlet of the aquifer and for different dispersion parameters considered. The impact of the dispersion coefficient varying with respect to the depth of pollutants filtration in the sub-surfaces has been evaluated. It has been observed that the concentration of pollutants in the aquifer is influenced by various boundary conditions. The dispersion parameter b has an important effect on the transport of solutes in the aquifer more than the parameter, a. In other words, the mass of the retained solute increases with parameter, b. Nevertheless, in experimental conditions, some factors such as pH and temperature could have significant effects on the transport through porous columns. These conditions will be considered in future works.

## Conclusion point-wise

• The effect of dispersion, function of the distance of the transport of solutes in a porous medium has been analyzed for different boundary conditions at the outlet of the aquifer and for different dispersion parameters considered.
• It has been observed that the dispersion parameter b has an important effect on the transport of solutes in the aquifer more than the parameter, a.
• The mass of the retained solute increases with parameter, b.
• Future works is focused on some factors such as pH and temperature to evaluate their effects on solute transport through porous columns.

## Declarations

### Author contribution statement

Calvia Yonti Madie: Performed the experiments; Contributed reagents, materials, analysis tools or data; Wrote the paper.

Fulbert Kamga Togue, Paul Woafo: Conceived and designed the experiments; Analyzed and interpreted the data.

### Funding statement

This research did not receive any specific grant from funding agencies in the public, commercial, or not-for-profit sectors.

### Data availability statement

Data will be made available on request.

### Declaration of interests statement

The authors declare no conflict of interest.

### Additional information

No additional information is available for this paper.
